# Hypersensitivity to Aspirin and other NSAIDs: Diagnostic Approach in Patients with Chronic Rhinosinusitis

**DOI:** 10.1007/s11882-015-0552-y

**Published:** 2015-07-07

**Authors:** Joanna Makowska, Anna Lewandowska–Polak, Marek L. Kowalski

**Affiliations:** Department of Immunology, Rheumatology and Allergy, Healthy Ageing Research Center, Medical University of Łódź, 251 Pomorska Str., 92-213 Łódź, Poland

**Keywords:** Chronic rhinosinusitis, Aspirin, NERD, Drug challenges

## Abstract

Hypersensitivity to nonsteroidal anti-inflammatory drugs (NSAIDs) associated with chronic rhinosinusitis (CRS) and/or asthma comprises a distinct clinical syndrome referred to as NSAIDs exacerbated respiratory disease (NERD). Patients with NERD tend to have more severe course of both upper (CRS and nasal polyps) and lower airway (asthma) diseases and are usually recalcitrant to conventional treatment modalities. Diagnosing and phenotyping of patients with NERD are critical for prevention of drug-induced adverse reactions and open novel options for management of underlying chronic airway inflammatory diseases. Diagnosis of NERD is based on detailed clinical history confirmed by challenge with aspirin, but new diagnostic approaches are currently being developed. This review article focuses on the diagnostic approach to a patient with CRS and hypersensitivity to NSAIDs, emphasizing the importance of diagnosis for proper patient’s management.

## Introduction

Acute forms of hypersensitivity to aspirin and other nonsteroidal anti-inflammatory drugs (NSAIDs) are among the most common drug hypersensitivities, may affect 1–2 % of general population and manifest with the whole variety of symptoms involving skin (rush, urticaria, and angioedema), respiratory tract (rhinorrhea, nasal congestion, and bronchospasm), and in some patients systemic anaphylaxis may develop [1, 2•]. Among patients with asthma and/or chronic rhinosinusitis (CRS) with nasal polyps, the prevalence of NSAIDs hypersensitivity is significantly higher reaching 26 % and is associated with severe eosinophilic hyperplastic inflammation of both upper and lower airway mucosa [3]. This syndrome has been previously referred to as “aspirin triad”, and more recently, the term NSAIDs-exacerbated respiratory disease (NERD) has been used, reflecting the presence of chronic inflammation of upper and lower mucosa of patients with this type of drug hypersensitivity [4•].

## Chronic Rhinosinusitis and NSAID-Hypersensitivity

**Fig. 1:**
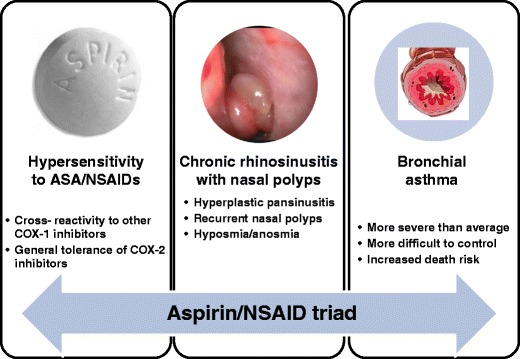
Clinical characteristics of NSAID exacerbated respiratory disease (modified from M.L .Kowalski, S. Bavbek, Aspirin Exacerbated Respiratory Disease, in Global Atlas of Asthma, Eds. C.A. Akdis, I. Agache, pp 92-94, EAACI 2013)

History of hypersensitivity to aspirin and other NSAIDs is a hallmark of particularly persistent and resistant to treatment form of rhinosinusitis, associated with recurrent nasal polyposis [5–8] (Fig. 1). Although NSAIDs can evoke hypersensitivity reactions in patients with CRS, but without lower airway involvement, the majority of patients would suffer from chronic bronchial asthma. The higher than usual severity of the upper airway disease in NERD patients is reflected by recurrence of nasal polyps and frequent need for sinus surgery [7, 9]. As it has been documented with computer tomography in these patients, mucosal hypertrophy usually involves all sinuses and nasal passages and has significantly higher extent and thickness compared to CRS in aspirin tolerant patients [10].

## Pathophysiology of NSAIDs-Exacerbated Respiratory Disease

Pathophysiology of NERD should be considered as a coincidence of two possibly distinctive, but to some extent overlapping mechanism: one being responsible for NSAIDs-induced acute hypersensitivity reaction and the other determining the presence of chronic eosinophilic mucosal inflammation in the airways, and resulting in persistent symptoms of rhinosinusitis, nasal polyps, and asthma [11].

### Mechanism of Acute NSAIDs-Induced Hypersensitivity Reactions

Hypersensitivity reaction induced by aspirin and NSAIDs is not immunological phenomenon, but results from inhibition of cyclooxygenase-1 and prostaglandin synthesis, which leads to activation of inflammatory cells (mostly mast cells and eosinophils, but possibly platelets) with subsequent release of leukotrienes and other inflammatory mediators in the mucosa [12–14]. These mediators are responsible for clinical manifestation of acute hypersensitivity reaction: bronchial obstruction, rhinorrhea, and nasal congestion. Avoiding aspirin and other NSAIDs with strong anti-COX-1 activity or using selective COX-2 inhibitors would prevent development of acute reactions. Although the causal relationship between inhibition of COX-1 by NSAIDs and development of symptoms has been well documented, the mechanism of inflammatory cells activation by NSAIDs is speculative and potentially may involve increased susceptibility of cyclooxygenase-1 to inhibition by NSAIDs, intrinsic deficiency of prostaglandin E_2_ [15•], and/or decreased function of prostaglandin E_2_ [16, 17]. Cysteinyl leukotrienes (LTC_4_/D_4_) are consistently increased after positive aspirin challenge in lower and upper airways, and leukotriene LT_1_ receptor antagonists partially prevent NSAID-induced reaction suggesting a key role of leukotrienes in the pathophysiology of acute NSAIDs-induced reaction [18]. The role of other arachidonic acid metabolites as prostaglandin D_2_ (PGD_2_) or 15-hydroxyeicosatetraenoic acid (15-HETE) is also feasible [19–22, 23•].

### Pathomechanism of Chronic Eosinophilic Airway Inflammation

Increased tissue eosinophilia and release of eosinophil cationic protein (ECP) in nasal polyps from NERD patients have been linked to distinctive profile of cytokine expression and upregulation of cytokines related to eosinophil activation and survival (e.g., IL-5, GM-CSF, RANTES, and eotaxin) [16, 24, 25] and is associated with differential expression of remodeling markers [26]. It has been recently documented that higher INF-γ levels are produced by eosinophils in nasal polyps tissue from NERD patients and INF-γ promoted maturation of eosinophil progenitors, results in enhanced expression of eosinophil-associated genes related to the leukotriene pathways (e.g., CysLT_1_ receptor or LTC4 synthase) [27].

Mast cells seem to be important component of the upper and lower airway inflammation in NSAIDs-hypersensitive patients [28, 29], since serum baseline levels of mast cell derived mediators tryptase and stable PGD_2_ metabolite are elevated [30]. In the nasal polyp tissue, the density of mast cells (and stem cell factor) was correlated with the number of polypectomies, implicating an important role for these cells in the pathogenesis of nasal polyposis [28].

Several abnormalities related to both cyclooxygenase and lipoxygenase pathways of arachidonic acid (AA) metabolism have been documented in the upper airway mucosa of NERD patients [31, 32]. Decreased expression of COX-2 mRNA leading to lower generation of PGE_2_ by nasal polyps, nasal polyp epithelial cells, and bronchial fibroblasts was reported [33–36] which together with reduced expression of prostaglandin EP_2_ receptors could result in impaired anti-inflammatory response [37, 38]. An increased generation of cysteinyl leukotrienes, overexpression of enzymes involved in production of leukotrienes (5-lipoxygenase and leukotriene C4 synthase) together with increased expression of leukotriene type 1 (LT_1_) receptors in the nasal mucosa of NERD patients may result in local hyperresponsiveness to leukotrienes in this subpopulation of patients [39, 40]. On the other hand, chronic treatment with LT_1_ receptor antagonist is not more effective in relieving nasal and bronchial symptoms or reducing polyp size in NERD as compared to NSAID-tolerant patients, thus other factors had to be involved in the persistence of inflammation in these patients [41, 42]. A decreased production of the anti-inflammatory lipoxin A_4_ in nasal polyp tissue and peripheral blood leukocytes from NSAID-sensitive patients further suggests an important role for dysregulation of AA metabolism [39, 40]. However, in patients with NERD eosinophilic inflammation in the lower and upper airways may precede, even several years, development of hypersensitivity to aspirin and persists even if they avoid intake of aspirin or other NSAIDs, pointing at involvement of other factors and mechanism, beyond AA metabolism [43].

The role for infections, both viral and bacterial, has been postulated, but not convincingly documented [44]. Higher concentrations of IgE-antibodies to *Staphylococcal aureus* enterotoxins (SAEs) in nasal polyps and serum have been associated with the presence of NSAIDs hypersensitivity, suggesting that *Staphylococcus aureus* superantigens may trigger T cell-mediated inflammatory reaction and/or exert direct effects on eosinophil proliferation and survival in the airway mucosa of NERD patients [45, 46]. Genetic background may be also important factor determining different pathophysiology and higher severity of CRS in NSAIDs hypersensitive patients [47].

## Diagnostic Approach to a Patient with NERD

**Fig. 2:**
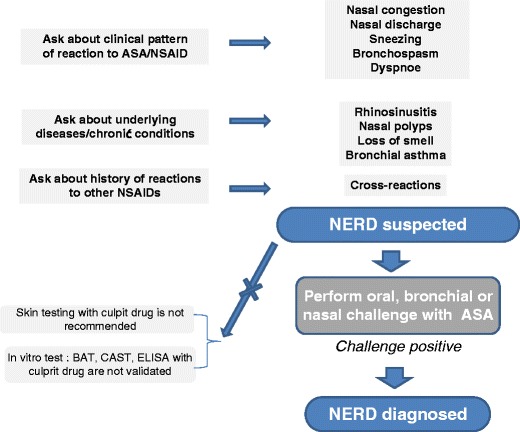
Diagnostic steps in a patient with chronic rhinosinusitis and suspected hypersensitivity to NSAIDs

Patients suspected to have NERD require not only documentation of an acute hypersensitivity reaction (by history and/or aspirin challenge) but also detailed evaluation of the extent of underlying diseases of the upper and lower airways (Fig. 2).

### Diagnosis of Chronic Rhinosinusitis

Diagnosis of CRS is based on history of presence of typical sinonasal symptoms (nasal blockage or obstruction, nasal discharge, and olfactory dysfunction) for more than 12 weeks and should be supported by nasal endoscopy and computed tomography (CT) scan of paranasal sinuses [48, 49]. Patients with NSAIDs hypersensitivity on average would have a history of long-lasting CRS with higher than average severity and resistance to both pharmacological and surgical treatment [7]. Reduced or lost sense of smell which commonly occurs in CRS patients with nasal polyps with and without NSAIDs hypersensitivity may be a leading symptom in NERD patients [50].

A distinctive feature of CRS in NERD patients is rapid recurrence of nasal polyps and mucosal hypertrophy following standard polypectomy or even functional endoscopic sinus surgery (FESS) [9]. It has been documented that patients with NERD have ten times increased risk of polyp recurrence after FESS as compared to aspirin tolerant patients [48, 49].

On CT scans, almost all patients with NERD have mucosal hypertrophy, and its extent is significantly higher in NSAIDs-hypersensitive as compared to NSAIDs-tolerant patients [10]. The intensity of sinus hypertrophy assessed by CT may predicts probability of NERD, and sinus CT score below 12 would support the likelihood of aspirin tolerance in a patients with unclear history of hypersensitivity reaction to aspirin and NSAIDs [48].

### Comorbidities

Only a tiny fraction of patients with CRS and nasal polyps is reacting to aspirin and NSAIDs only with upper respiratory symptoms, and even those with time will present lower symptoms after NSAIDs. Large majority will have a history of lower airway symptoms (dyspnea and wheezing) after aspirin intake, and these patients usually suffer from chronic bronchial asthma [51]. Patients with NERD tend to suffer from more severe form of the disease which is associated with less control and with increased risk of life-threatening asthma attacks [8, 2•].

All patients with nasal polyps and NSAIDs hypersensitivity should also undergo full allergic evaluation since majority (50–70 %) may have allergic sensitizations to inhalant allergens; thus, atopy should not exclude the suspicion of NSAIDs hypersensitivity if other risk factors (e.g., severe asthma or nasal polyposis) exist [52–55]. The presence of atopy was suggested to be a risk factor for aspirin hypersensitivity among asthmatics patients challenged with oral aspirin, thus atopic sensitization to inhalant allergens may be important mechanism contributing to the pathogenesis of the airway inflammation in a patient with NERD [53].

## Diagnosis of NSAIDs Hypersensitivity

### History and Physical Examination

Patient with NERD would present a history of acute rhinorrhea and nasal congestion usually accompanied by bronchial symptoms (dyspnea), which develop usually within 1–2 h after ingestion of aspirin or other NSAIDs (e.g., naproxen, diclofenac, or ketoprofen) with known COX-1 inhibitory capacity. On the other hand patient usually reports, that some NSAIDs, which are weak inhibitors of prostaglandin synthesis, like paracetamol and preferential COX-2 inhibitors, are well tolerated.

Approximately 10 % of patients with NERD may simultaneously manifest non-respiratory, usually cutaneous symptoms (urticaria and/or angioedema) after intake of aspirin. Thus, a patient with CRS and history of adverse reaction to aspirin or other NSAIDs should be fully evaluated with respect to potential type of hypersensitivity which may involve in addition lower respiratory and cutaneous symptoms [2•].

### Provocations Tests

Although in clinical practice diagnosis of drug hypersensitivity is usually based on history of adverse reaction associated with the culprit drug, such history may not be reliable leading to either under diagnosis or over diagnosis of drug hypersensitivity [56•]. In study of Dursun et al. [57], history of NSAIDs-induced reactions could not be confirmed with oral challenge in 16 % of patients with NERD, and only 43 % patients with chronic sinusitis, nasal polyps, and asthma who were avoiding aspirin or NSAIDs had a positive oral aspirin provocation. Thus, oral aspirin challenge is recommended to confirm the diagnosis of NSAIDs hypersensitivity regardless of the clinical manifestation, while nasal or bronchial provocation with lysine-ASA may be alternatively used in patients with respiratory symptoms [58, 59].

**Fig. 3:**
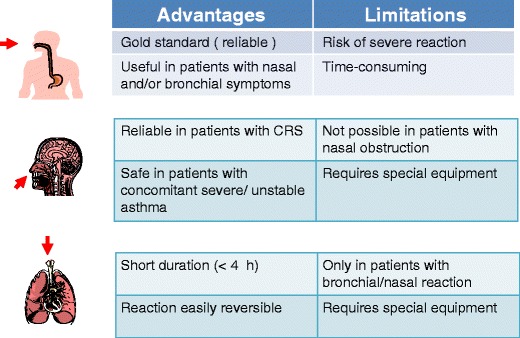
Advantages and limitations of oral, intranasal, and bronchial route of aspirin provocation in patients with NERD (modified from M.L. Kowalski In vivo diagnosis of NSAIDs hypersensitivity, in Global Atlas of Allergy, Eds C. A. Akdis I. Agache, pp. 158-160, EAACI, 2014)

Advantages and limitations of various provocation methods are summarized in Fig. 3.

### Oral Provocation Test

Several protocols for oral aspirin provocation varying in recommended aspirin dose increments and intervals between up dosing are available, and recently, EAACI/GA_2_LEN expert panel proposed a protocol which merges experience of several groups [58, 60–62].

Oral challenge should be performed in a setting with immediate access to emergency equipment, and service after all contraindications were considered [63]. In patients with asthma, the disease should be well controlled and FEV_1_ should exceed 70 % of predicted values. On the first day, in order to establish baseline variability, placebo capsules are administered every 1.5–2 h and FEV_1_ is measured every 30 min (respiratory function is monitored even if in patients without history of asthma). On the second day, patient receives initially 10–30 mg of aspirin and the dose is doubled in 1.5 to 3 h intervals until positive reaction occurs. In a patient with rhinosinusitis, without bronchial asthma, development of upper respiratory symptoms (nasal congestion and rhinorrhea) may be diagnostic. Acoustic rhinomanometry can be used to document nasal obstruction during the challenge. In a patient with asthma, the reaction is considered positive if at least 20 % fall in FEV_1_ occurs, which may be accompanied nasal, ocular, cutaneous, or gastrointestinal symptoms. If the final dose 312 mg of aspirin (corresponding to 500 mg of cumulative dose) is ingested and well tolerated the patient is considered to be aspirin tolerant [63].

The negative challenge result should be analyzed with caution since it may reflect either development of “silent desensitization” during the challenge procedure or blocking effects of medications used to control chronic inflammatory disease [64, 65]. For example, the use of antihistamines may significantly decrease development not only of cutaneous but also respiratory symptoms. On the other hand, discontinuing controller medications before aspirin challenge in patients with suspected NSAIDs hypersensitivity is not recommended since it may increase the risk of severe reactions [66, 67].

Since diagnostic oral challenge tests are time-consuming procedure, requiring well-experienced personnel and may be associated with a risk of severe systemic reaction, oral provocation test may be substituted with inhaled or intranasal aspirin challenges [58, 68].

### Nasal Provocation Test

Intranasal provocation with lysine aspirin (a soluble form of ASA) is a good alternative for oral challenge since it is rapid, safe, and can be performed in an outpatients setting and even in asthmatic patients with low pulmonary function not suitable for bronchial provocation [5, 68–72]. However, this route of challenge cannot be used in patients with significant nasal obstruction, turbulent nasal flow, or unspecific nasal responsiveness [68]. Table 1 presents studies reporting diagnostic effectiveness of intranasal challenges with aspirin. The sensitivity of aspirin nasal challenge test ranges from 38 % in study of Patriarca [72] to 87 % in study of Milewski [68] and specificity from 84 [71] to 96 % [68]. The study of Miller [73] in which higher doses of lysine aspirin showed similar sensitivity 88 %. The nasal provocation test seems to be a safe alternative for oral and bronchial challenge in patients with severe asthma as in most patient intranasal delivery of aspirin did not evoke severe bronchoconstriction [68, 74•, 75•]. However, in occasional, patients application of lysine aspirin by atomizer or using of higher doses of aspirin can lead to bronchial symptoms with significant drop in FEV_1_ > 30 %, which is pointing at necessity of respiratory function monitoring by spirometry during nasal provocation with aspirin [70, 73].

**Table 1:** Diagnostic performance of nasal provocations with soluble NSAIDs (lysine-aspirin or ketorolac) in patients with NERD. PNIF-peak nasal inspiratory flow, ATA aspirin tolerant asthma, N/A not available

Author	Reference number	Year	Drug	No of NERD patients	Dose of Lysine-ASA/ketorolac administered	Method for assessment	Sensitivity	Specificity	PPV	NPV	Comments
Patriarca G	70	1991	lysine aspirin	45	30 mg	Symptoms, PNIF	37.80 %	92.10 %	N/A	N/A	
Pawlowicz A	68	1991	lysine aspirin	8	20 mg	FEV_1_, nasal symptoms	N/A	N/A	N/A	N/A	Delivery of ASA by atomizer, ASA could reach lower airways, 100 % had drop of FEV_1_ > 15 %, one patient had urticaria
Wellebrock M	69	1993	lysine aspirin	153	10 mg	Nasal symptoms, PNIF	67 %	84 %	N/A	N/A	Two patients had bronchial symptoms
Milewski M	66	1998	lysine aspirin	51, 10 healthy controls and 13 with ATA	16 mg	Rhinomanometry	86.70 %	95.70 %	97.20 %	78.60 %	Ten drop outs due to total obstruction or marked fluctuation in nasal flows
Casadevall J	5	2000	lysine aspirin	15, 8 ATA and 8 healthy	25 mg	Acoustic rhinometry	73 %	94 %	N/A	N/A	
Alonso-Llamazares A	67	2002	lysine aspirin	20, 10 ATA asthmatics, 30 healthy	18 mg	Rhinomanometry	80 %	92.50 %	84.20 %	89.20 %	
White A	65	2006	ketorolac	18	7,8 mg		78 %	64 %	N/A	N/A	
Muñoz-Cano R	72	2011	lysine aspirin	72	29 mg to each nostril	Acoustic rhinometry and symptoms score		N/A	N/A	N/A	No pulmonary or systemic reactions
Wismol P	73	2012	lysine aspirin nasal challenge	30	16 mg		93,3 % in NERD	N/A	N/A	N/A	
Celikel S	74	2013	ketorolac	165		PNIF	69 %	N/A	N/A	N/A	16 % had mild lower respiratory symptoms, two severe bronchospasm, 5 % urticaria/angioedema
Miller B	71	2013	lysine aspirin	131	40 mg	Acoustic rhinometry and PNIF	88 %	N/A	N/A	N/A	Acoustic rhinometry more sensitive than PNIF

Lysine aspirin can be substituted with ketorolac another NSAID, which in a soluble form is more easily available in some countries [76]. Reproducibility of nasal challenges in which combination of acoustic rhinomanometry and symptoms scores is used to assess the results [73, 74•, 77] is very high reaching 98 % [73]; however, good reproducibility of peak nasal inspiratory flow (PNIF) measurements have been also reported [78].

### Bronchial Aspirin Provocation

Bronchial provocation with lysine aspirin is reliable alternative for oral provocation with aspirin in patients with diagnosis of asthma or with history of lower respiratory symptoms after NSAIDs [58, 63]. However, in patients with CRS without asthma or with history of only upper airway symptoms after NSAID, oral provocation cannot be substituted with inhalation challenge.

## In Vitro Tests for Diagnosis of NSAIDs Hypersensitivity

Although several tests based on measuring of in vitro activation of peripheral blood leukocytes have been proposed, no in vitro test can match provocation challenges with respect to sensitivity or specificity (Table 2).

**Table 2:** Performance of in vitro test in the diagosis of NERD

First author	Year	Test	No of patients	Cells/stimulus	Sensitivity	Specificity	PPV	NPV
Kowalski ML	2003	AspiTest (15-HETE release)	16	Leukocytes/ 200 uM of ASA	–	–	–	–
Kowalski ML	2005	AspiTest (15-HETE release)	43	Leukocytes/ 200 uM of ASA	82 %	83 %	0.79	0.89
Kosec P	2011	AspiTest (15-HETE release)	26	leukocytes / 500uM of ASA	63 %	50 %	–	–
James A	2013	15-HETE release from eosinophils upon stimulation with ASA	7	Eosinophils / 200uM of ASA	–	–	–	–
Lebel B	2001	CAST	55	Leukocytes/beta-lactam, ASA, paracetamol				
Bavbek S	2009	CAST	30	Basophils/ASA, diclophenac	25 %	92.30 %	0.29	0.91
de Weck	2009	CAST+BAT	152 patients + 165 controls	Leukocytes/basophils/SA/paracetamol/naproxen/metamizol/diclophenac	65 %	80 %	–	–
Sanz ML	2005	CAST+BAT	60	Leukocytes/basophils/ASA/paracetamol/naproxen/metamizol/diclophenac	73.30 %	71.40 %	–	–
Gamboa P	2004	BAT (CD63)	60	Basophils/ SA/paracetamol/naproxen/metamizol/diclophenac	63.30 %	93.30 %	–	–
Sanz ML	2005	BAT	60	Basophils/paracetamol/naproxen/metamizol/diclophenac	66.70 %	93.30 %	–	–
Celik G	2009	BAT (CD63)	10	Basophils	30 %	40 %	–	
		BAT (CD203)	10	Basophils	70 %	45 %		
		BAT (CD69)	10	Basophils	80 %	34 %		
BavbekS	2009	BAT (CD63)	18	Basophils/ASA, diclophenac	16.70 %	91.70 %	–	–
		BAT (CD203)		Basophils/ASA, diclophenac	22 %	100 %	–	–

### Sulfidoleukotrienes Release Assay

Aspirin-triggered release of LTC4 from peripheral blood leukocytes (CAST-ELISA) has been tested in several studies [79–81], in highly selected population, but has not been validated for routine use [82–84].

### Basophil Activation Tests (BAT)

BAT was tested in populations of patients with both respiratory and/or cutaneous type of hypersensitivity to different NSAIDs with not well characterized, control populations, and its utility for diagnosis of respiratory type of aspirin sensitivity has not been sufficiently documented [75•, 85–87].

*15-HETE generation assay (ASPITest)* is based on observation that aspirin could more readily trigger in vitro generation of 15-hydroxyicosatetraenoic acid (15-HETE) from nasal polyp epithelial cells and peripheral blood leukocytes from ASA-hypersensitive patients, as compared to ASA-tolerant asthmatics or healthy subjects, and measurement of 15-HETE release from PBLs has been proposed to be used to confirm history of aspirin hypersensitivity in patients with NERD [19, 20, 88]. The sensitivity of aspirin-triggered 15-HETE release (ASPITest) tested in highly selected population of patients with NERD demonstrated 82 % sensitivity and 83 % specificity [88]. However, more recent studies including more heterogeneous populations of patients with asthma and/or using isolated eosinophils demonstrated non-specific release of 15-HETE form PBLs and could not reproduce diagnostic performance of 15-HETE measurement for NERD [89, 90]. Further studies are required to refine the methodology and to assess diagnostic effectiveness of this method.

## New Diagnostic Approaches

Several biochemical abnormalities related to arachidonic acid metabolism can be detected in easily available biological materials like blood, urine, or exhaled air of patients with NERD. However, diagnostic value of measurement of AA metabolism products to predict or confirm NSAIDs hypersensitivity has not been assessed.

### LTE_4_ Urinary Levels

Increased basal levels of leukotriene E_4_ in urine have been consistently reported in NERD patients, but a significant overlap with non-sensitive asthmatics was observed in most studies, not allowing for using a single urine LTE_4_ measurement to predict ASA-sensitivity [91–93].

### Eicosanoid Profile in Exhaled Breath Condensates (EBC)

Sanak et al. proposed measuring a set of 19 eicosanoids in EBC by complementary high-performance liquid chromatography and/or gas chromatography–mass spectrometry to distinguish ASA-tolerant from ASA-sensitive asthmatics [94]. The eicosanoid profiling in EBC allowed for 92 % correct classification of aspirin-intolerant subjects. The practical use of eicosanoids measurements in EBC to diagnosis of CRS with ASA-sensitivity is to be assessed.

### Genetic Determinants of NSAIDs Hypersensitivity

Several genes have been associated with aspirin hypersensitivity—most are related to arachidonate metabolism or inflammatory pathways [47, 95]. HLA-DPB1*0301 allel has been associated not only with aspirin-hypersensitivity but also with higher prevalence of CRS in NERD patients [96–98]. A genome-wide association study documented an increased risk for developing aspirin hypersensitivity in adult patients and two SNPs located on chromosome 6, and one of them (rs3128965) was identified as a genetic marker for NERD [99].

## Conclusion

Hypersensitivity to aspirin and other NSAIDs is a hallmark of severe chronic upper and lower airway disease, thus should be suspected and carefully diagnosed in patients with CRS. Oral aspirin challenge remains a gold standard for diagnosing aspirin sensitivity; however, intranasal challenge with soluble form of aspirin may be a diagnostic alternative. Phenotyping of NERD allows to recommend well-tolerated NSAID, if analgesic and anti-inflammatory therapy is need. For some patients with confirmed hypersensitivity to NSAIDs, aspirin after desensitization may be a valuable option for management of CRS. Further work is necessary to understand the pathomechanism of this syndrome and to improve the diagnosis of NERD.
